# The impact of adolescents’ voice through an online school radio: a socio-emotional learning experimental project

**DOI:** 10.3389/fpsyg.2023.1197193

**Published:** 2024-09-06

**Authors:** Patrícia Sarmento, Mafalda Lobo, Kalpna Kirtikumar

**Affiliations:** Semear Valores Cooperative, Cascais, Portugal

**Keywords:** socio-emotional skills, online school radio, experimental project, adolescents, virtues and character strengths model, voice, citizenship

## Abstract

Universal school-based socio-emotional learning (SEL) programs for adolescents have shown their efficacy in producing positive outcomes. The aim of the current study is to present an original school-based program and project for adolescents—*Semear Valores On-air* – and to assess the relationship between participation in the project and students’ socio-emotional skills. Based on the character strengths and virtues model, this online school radio project aimed at promoting communication, creative thinking, adaptability, and resilience skills in adolescents and giving them the opportunity to become influential agents of well-being and citizenship. As part of the school curriculum, students were invited to create and record radio shows and podcasts. An online school radio was thus created, and it continues to broadcast all over the world, with music, daily shows, and interviews 24/7. It was developed within the framework of the Gulbenkian Academies for Knowledge, a nationwide Portuguese program, that seeks to prepare children and youth for change, to enable them to deal with complex problems, and to expand their opportunities for achievement. A quasi-experimental design, with a mixed qualitative-quantitative approach was used to analyze data collected from 112 adolescents in the second year of its implementation, in 2020–2021. Results suggest that (1) teachers’ perceptions of student’s socio-emotional skills in the post test showed more positive associations with the participation in the project, than participant’s perceptions; (2) students identified eight types of lessons learned, the one most referred was the improvement of socio-emotional skills and learning about themselves; and (3) the combined opportunities for adolescents to learn more about themselves, to express themselves and to practice socio-emotional skills are important ingredients for their motivation and active engagement in the project. Overall, these results indicate that participation in the project is associated with positive outcomes for the adolescents and that both monitoring and evaluation data are very important to interpret the outcomes in a more comprehensive manner.

## Introduction

Research has consistently shown the importance of implementing universal approaches to foster socio-emotional skills in adolescents (Durlak et al., 2011; Taylor et al., 2017; Mahoney et al., 2018). Socio-emotional skills include an individual’s attitudes, internal states, approaches to tasks, management of behavior and feelings, and beliefs about the self and the world (OECD, 2021). The process by which children and youth develop these skills is named Social and Emotional Learning (SEL; Elias et al., 1997, p. 2).

The most widespread SEL program is Social and Emotional Learning, developed by *Collaborative for Academic, Social and Emotional Learning*, which addresses Self-awareness, Self-management, Responsible decision making, Social awareness and Relationship skills (CASEL, 2015). In recent years, the *Organisation for Economic Co-operation and Development* (OECD, 2021) has contributed to the SEL field with a conceptual model based on the Big Five framework (Kankaraš and Suarez-Alvarez, 2019). This model addresses the domains of Open-mindedness; Task performance; Engaging with others; Collaboration and Emotional regulation, each of them including more specific skills (OECD, 2021). A recent study and carried out in different cities around the world, including the United States, Canada, Colombia, South Korea, Finland, Turkey, Russian Federation, People’s Republic of China, and Portugal (OECD, 2021), studied the relation between socio-emotional skills of children and adolescents and their school grades along with the scores obtained in a cognitive abilities test. Although the strength of the relations between certain socio-emotional skills and school grades was relatively weak, it was consistent (OECD, 2022). One of the frameworks that has been gaining considerable interest in the context of school-based SEL programs is the character strengths and virtues model (Peterson and Seligman, 2004). This model addresses 24 character strengths (e.g., Curiosity, Love, Teamwork, Prudence, Persistence), which are expressed through thoughts, feelings, and behaviors and grouped into 6 virtues: Wisdom and knowledge, Courage, Humanity, Transcendence, Temperance, and Justice (Peterson and Seligman, 2004). According to the authors, the use of these strengths would contribute to a meaningful and pleasant life. Several character strengths are positively correlated with positive outcomes, such as decreased behavioral problems, better school performance and social functioning (Park and Peterson, 2009; Shoshani and Slone, 2013), decreased levels of stress, depression and anxiety (Park and Peterson, 2009; Gillham et al., 2011; Proctor et al., 2011a; Wood et al., 2011), improved well-being and greater satisfaction in life (Proctor et al., 2011a; Abasimi et al., 2017; Kretzschmar et al., 2023).

Meta-analysis studies on SEL programs applied to different school levels have demonstrated positive outcomes in enhancing overall well-being, encouraging prosocial behaviors, improving academic achievements, and decreasing both externalizing and internalizing problems (Durlak et al., 2011; Sklad et al., 2012; Taylor et al., 2017; van de Sande et al., 2019). Also, education projects based on the character strengths and virtues model have shown positive outcomes (Proctor et al., 2011b; Silva, 2013; Kern and Kaufman, 2017).

Socio-emotional skills can be shaped through learning (Kautz et al., 2014; Gueldner et al., 2020). According to the CASEL model, these skills can be learned through instruction, practice, and feedback (Gueldner et al., 2020). The way that school-based SEL programs are implemented is critical for their success (Durlak and DuPre, 2008; Durlak et al., 2011). Researchers have identified some critical components of implementation that matter the most when it comes to outcomes, notably, Dosage: *How much of the program is delivered?*; Fidelity: *In which degree is the program being followed?*; Adaptation: *What changes are made to the original program?*; Quality of delivery: *How well is the program conducted?* and Participant responsiveness: *To what degree are the participants actively involved*? (Durlak, 2016). Evidence suggests that the level of implementation achievement is one of the most important factors affecting program outcomes (Durlak, 2016). A systematic review of 41 school-based mental health intervention studies found that 36% of the time, these critical components of implementation were positively associated with student outcomes (Rojas-Andrade and Bahamondes, 2019).

Historically, despite many SEL programs strongly focusing on learning and practicing socio-emotional skills (Taylor et al., 2017; Mahoney et al., 2018), they lack a community give-back component. In recent years, some SEL projects have stimulated children and youth’s skills, by providing opportunities for active civic participation in their communities (Branquinho and Matos, 2016). Projects in which adolescents are active agents of change seem to contribute positively to socio-emotional development (Frasquilho et al., 2018). Dobia et al. (2020, p. 178) recommend a “greater emphasis on student voice and agency” for a more successful SEL implementation in secondary schools. Fewer projects have used radio as an instrument of youth participation and/or to develop socio-emotional skills (Jaime-Osorio et al., 2019; Ballinas-Gonzalez et al., 2020). In one of these studies, students were shown to have improved their oral and conversational skills, as well as their relationships (Jaime-Osorio et al., 2019). Since 2015, SEL has regaining importance in Portuguese Education. The Ministry of Education has adopted a humanistic framework, that reintroduced citizenship education into the curriculum and set expectations for students to develop socio-emotional skills.

This paper aims at presenting the *Semear Valores On-air* Academy, a project that was implemented for a 3-year period (2019–2022) in a Portuguese public school, involving 10 teachers and 249 students. It took place under the *Gulbenkian Academies for Knowledge* initiative, which supported more than 100 SEL projects – called “academies.” This initiative adopted the OECD evaluation framework for socio-emotional skills. Based in the character strengths and virtues model (Peterson and Seligman, 2004), *Semear Valores On-air* challenged students to develop their socio-emotional skills and to create an online school radio. The project thus pushed students to use their voice as a positive influence, impacting their communities. Another goal is to understand the relationship between the participation in the academy and students’ socio-emotional skills.

## Description of the academy *Semear Valores on-air*

### Pedagogical framework(s), and principles

The character strengths and virtues model (Peterson and Seligman, 2004) was used to foster a collaborative atmosphere and instill a sense of well-being and resilience. We invited students to recognize and appreciate the character strengths in themselves and in their colleagues and to intentionally use them in their day-to-day lives. Also, it served as the main theme for the radio scripts. Whatever theme students chose, they should look for character strengths (e.g., when talking about the soccer championship, they would discuss the teams’ strengths; in an interview, they would ask a question about the interviewee’s strengths).

Given the positive impact of active methods and practical approaches in promoting socio-emotional skills (CASEL, 2015; World Economic Forum, 2016), we adopted a project pedagogy focused on creation (Figueiredo, 2017) combined with group dynamics.

We established the following guiding principles and prerequisites:

1. The academy should be part of the students’ curriculum and not an extracurricular activity. This maximizes students’ attendance and promotes interdisciplinarity with other school subjects.
2. The class director (i.e., the teacher who is responsible for a particular class in school) must be motivated to participate.
3. Class directors collaborate with the academy’s facilitators: they participate in the sessions, arrange the necessary spaces, give feedback, and are involved in the evaluation process.
4. Class directors must attend an initial training.
5. Students are encouraged to try different roles (e.g., radio announcer), different program formats and to continuously improve their work. This entails a different mindset from the one required by most of the pedagogical assignments. The grade is not the ultimate goal. They must improve their scripts and practice orally before recording. This implies to be open to feedback and go the extra mile to improve.
6. Schools must provide appropriate rooms for different sessions: studio and classrooms with computers.

### Objectives, pedagogical format and implementation

Our academy proposes a creative curriculum to promote students’ socio-emotional skills, namely, communication, creative thinking, adaptability, and resilience, while empowering them to be active citizens.

The program curriculum was designed for one school year. The academy was implemented in a Portuguese school, from 2019 to 2022 in the Citizenship class, by two facilitators and two radio editors, all part-time workers. The first step was to get the studio and the equipment ready. The school appointed a project coordinator, who helped the team by selecting the participating classes, announcing the training to teachers, and booking adequate rooms.

**Figure 1:**
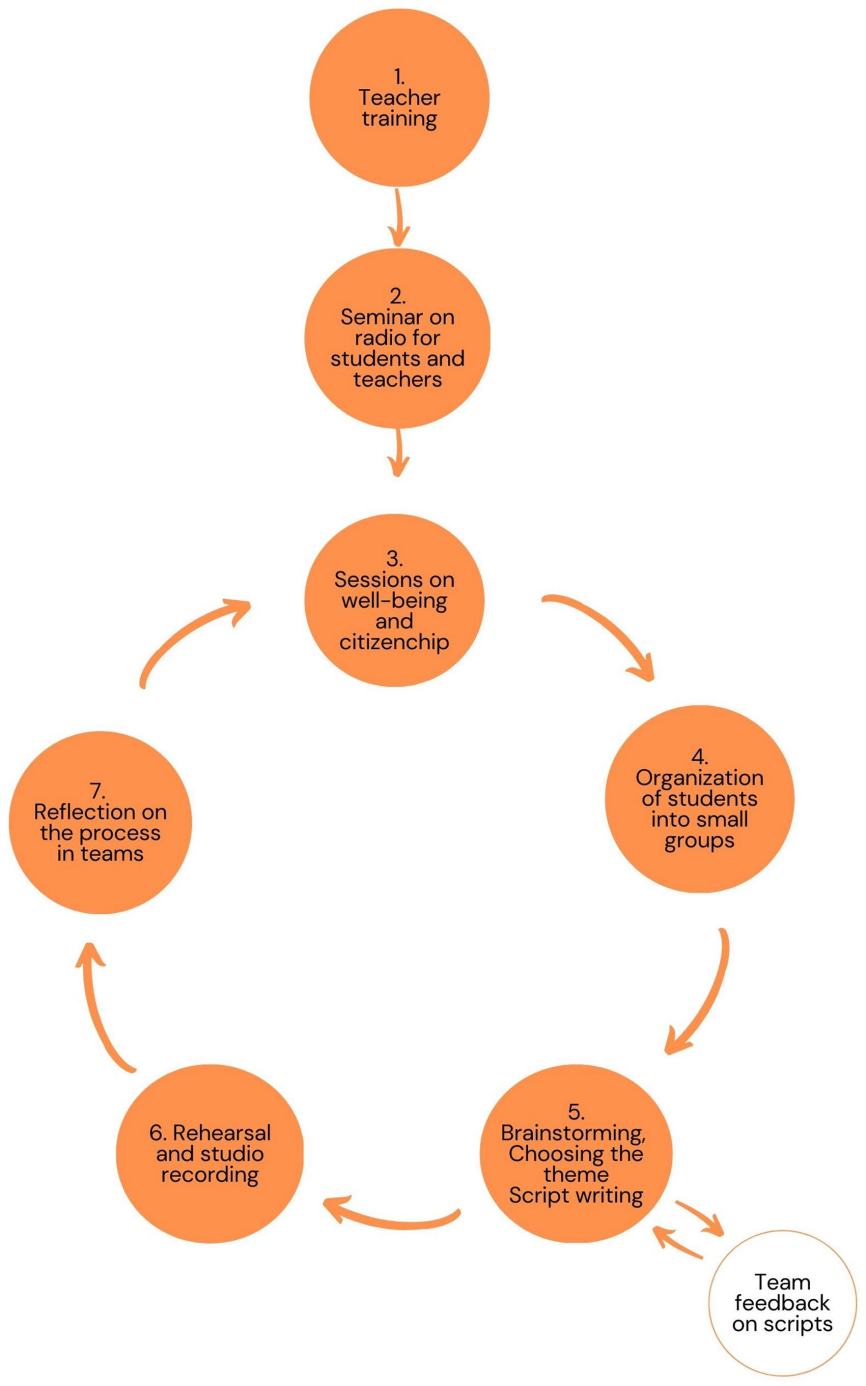
Stages of the academy.

As one can see in Figure 1, point 1, after selecting the classes, a four-hour training was built to introduce teachers to the academy and to the character strengths and virtues model. This training was mandatory for class directors and open to other school teachers. The first session was a seminar (point 2) which aimed to introduce the academy; to talk about the influence of radio worldwide; teach about the areas of radio (animation, programming and production) linked to different professions and raise awareness of the importance of communicating well. In the following three sessions (point 3, Figure 1), we collected the participants’ data, introduced the 24 character strengths and invited participants to look at their own strengths and at their colleagues’, through active methods. Students were organized into small groups (point 4) and each group chose a radio show format (point 5): doing an interview or talking about a subject. Afterwards, they had to choose the interviewee or the theme of the show (e.g., a film review, a biography). Then, groups researched on the topics selected. The process of script writing was fluid, and this was explained to students early on. After the first version of the script was completed, the facilitators reviewed it and offered their suggestions. Feedback was essential for improving the scripts. When the scripts were ready, students rehearsed their lines, and a time was scheduled to record in studio (point 6, Figure 1). If the selected format was an interview, they had to arrange a time with the guests to record it. Depending on the maturity of the group, the scheduling could be intermediated by the team. The moment of recording was one of great excitement and some nervousness too. At this stage, it was important to calm the students down. Finally, each group was invited to reflect on how the whole group work process went (point 7). Throughout the school year, the groups had the opportunity to go through the entire process two to four times, depending on the efficiency of each group.

We expected that different types of sessions would develop different socio-emotional skills: (1) the script writing sessions would foster creative thinking and communication, (2) the studio recording sessions would promote communication and adaptability, and (3) the well-being sessions and the academy format itself would foster resilience, because it would take effort and time until the radio shows were ready to go on air.

The radio shows were professionally edited by the communication partner, which was also responsible for creating the online radio, named by the first year’s participants as “MEGA Ibn radio.” This partner also created a podcast channel with all the shows produced. To create a greater sense of belonging to the radio, each class chose a name for the playlist, where all their programs sat. Both the online radio and the podcast channel were launched during the first year of the academy. On that day, all school listed to the radio launch. The online radio was broadcasted *via* internet to the world, 24/7 and APPs and the podcasts were broadcasted through Soundcloud. The broadcast routine included music and 3 daily shows.

The academy involved 10 classes from the 7th to the 12th grade. Some changes were made to the original plan, mainly because of the pandemic, the confinement and the lessons learned from the first year of the academy’s implementation. For instance, the format of the sessions changed from face-to-face to remote, in the periods of confinement. In year 1 and 2 the well-being sessions were adapted to address the negative effects of the pandemic and confinement, by adding themes such as emotional expression and management, and promoting positive relationships. From learnings made during the first year, other changes were implemented: (a) a higher number of script writing lessons; (b) the criteria for selecting students (minimum 9th graders, once they were more fluent in writing); (c) use of more active methodologies; (d) reduced number of participants per working group and (e) opportunities for students to try out radio sound design.

We will present only the data from the second year of the academy’s implementation, because the 1^st^ year was a pilot and in the 3^rd^ year data from teachers from the comparison group was missing.

## Methods

### Participants

In year 2, 84 students participated in the academy (intervention group, IG) and 54 composed the comparison group (CG). 61 (70.9%) attended the 9^th^ grade, 25 (29.1%) the 11th grade. Half were female and half male, 83.7% were Portuguese and 8.1% were migrant students and their average age was 15.7 years old. Of the 54 students in the comparison group, 30 attended the 9th grade (55.6%) and 24 (44.4%) the 11th grade. 44.4% of the students were female and 50.0% male (5.6% did not answer), 92.6% were Portuguese and 1.9% migrants. Their average age was 15.2. The intervention group (*N* = 84) is statistically different from the comparison group (*N* = 54) in the variables youth’s age, year of schooling (and others analyzed), so the results should be interpreted with caution.

### Design, procedure and measures

This study used a quasi-experimental single-group design with a mixed qualitative-quantitative approach (Euzébio et al., 2021).

With the help of the main teacher, we asked adolescents and their parents for informed written consent for data collection and for voice recordings, separately. We also informed the adolescents and families about the goals of the data collection and confidentiality terms. Subsequently, the students agreed to complete the instruments voluntarily in the classroom, under the supervision of the teacher and at least one member of the academy’s team. 138 students were invited to complete a socio demographic survey in September 2020, from which only 112 have done it. To assess students’ socio-emotional skills, we invited the same group and their main teachers to answer an online reduced version of the Survey on Social and Emotional Skills (*SSES*), by Kankaraš and Suarez-Alvarez (2019). As shown in Table 1, the reduced version was composed by 6 of the total 17 subscales of SESS: *cooperation* (to assess communication); *creativity* (creative thinking); *persistence* (resilience); *optimism*; *responsibility* and *curiosity* (adaptability). The adapted instrument for teachers was composed of 18 items (3 items per subscale) and for students, of 48 items (8 items per subscale). These data were collected between September and October 2020 (pre-test), and in June 2021 (post-test). 116 students completed the SESS questionnaire during class: 74 from the IG and 42 from the CG. To the students from the CG, it was offered the opportunity to engage in one radio show. From the five teachers that completed the SESS, data from one was removed because it was incomplete, so only answers from 4 were considered (2 teachers from the IG and 2 from the CG). To the teachers from the CG, it was offered the opportunity to participate in a training.

**Table 1:** Description of the socio-emotional skills evaluated and the correspondent SESS subscale and its description based on the assessment framework of Kankaraš and Suarez-Alvarez (2019).

Socio-emotional skill	Measured by the subscales (SSES)	Description (SESS)
Creative thinking	Creativity	“Generating novel ways to do or think about things through exploring, learning from failure, insight, and vision.”
Resilience	Persistence	“Persevering in tasks and activities until they get done.”
Communication	Cooperation	“Living in harmony with others and valuing interconnectedness among all people.”
Adaptability	Optimism	“Positive and optimistic expectations for self and life in general.”
	Responsibility	“Able to honor commitments and be punctual and reliable.”
	Curiosity	“Interest in ideas and love of learning, understanding and intellectual exploration; an inquisitive mind-set.”

For monitoring purposes, we assessed the program’s dosage; responsiveness, quality, and fidelity/adaptability, using different assessment tools (Durlak and DuPre, 2008; Alexandre et al., 2019). Dosage was assessed by observing and registering attendance in each session. Responsiveness was assessed through online satisfaction surveys aimed at students, teachers, and partners. The survey for students gathered data on satisfaction with the activities and lessons learned; the survey for teachers assessed their satisfaction with training (i.e., interest in the topic, clarity of presentation, methodology used, workshop’s relevance to the project, involvement of the participants); and finally, the survey for teachers and partners gathered information on the academy’s overall functioning and asked for suggestions. To measure the program’s quality, we used a criteria checklist, which included the verification of a set of conditions (e.g., training of facilitators; supervision/intervision; team meetings). Finally, fidelity/ adaptation was measured by verifying a checklist that measured the degree of completion of the planned sessions and adaptations.

### Data analyses

Statistical analyses were conducted using IBM SPSS Statistics 22.0. Descriptive statistics were run to analyze socio demographic data. A two-way ANOVA was conducted to evaluate the main effects of the group (i.e., intervention vs. comparison group) and time (i.e., pre or post-test) on students’ socio-emotional skills to determine if participation in the academy was associated with skills assessment. Pearson product–moment correlations were conducted to explore the relationships between dosage and each skill. Qualitative data analysis was explored with Excel, version 2,304, which allowed to organize students’ feedback from the sessions into categories (Campos, 2004).

## Results

### Results from students’ perceptions

Results indicated a significant interaction between the effects of time and group for Curiosity (*F*(1, 114) = 9.27, *p* = 0.003, partial Ƞ^2^ = 0.07) and Adaptability subscales (F(1, 114) = 4.09, *p* = 0.045, partial Ƞ^2^ = 0.03). Table 2 shows that for these skills, the students’ perceptions in the intervention group decreased in the post test (curiosity) or maintained (adaptability), while the students’ perceptions in the comparison group increased. There was no significant interaction between the effects of time and group for other skills.

**Table 2:** Descriptive statistics for SESS results, students’ version.

Socio-emotional skill	Group	*M*	SD
Creativity Pretest	Comparison Group	3.69	0.51
	Intervention Group	3.69	0.51
Creativity Post test	Comparison Group	3.80	0.53
	Intervention Group	3.67	0.55
Persistence Pretest	Comparison Group	3.76	0.57
	Intervention Group	3.76	0.63
Persistence Post test	Comparison Group	3.93	0.58
	Intervention Group	3.75	0.58
Cooperation Pretest	Comparison Group	4.16	0.39
	Intervention Group	4.27	0.47
Cooperation Post test	Comparison Group	4.14	0.41
	Intervention Group	4.16	0.53
Optimism Pretest	Comparison Group	3.70	0.77
	Intervention Group	3.80	0.72
Optimism Post test	Comparison Group	3.80	0.63
	Intervention Group	3.86	0.70
Responsibility Pretest	Comparison Group	3.79	0.44
	Intervention Group	3.74	0.55
Responsibility Post test	Comparison Group	3.96	0.43
	Intervention Group	3.84	0.50
Curiosity Pretest	Comparison Group	3.83	0.45
	Intervention Group	3.97	0.48
Curiosity Post test	Comparison Group	3.92	0.53
	Intervention Group	3.83	0.49
Adaptability Pretest	Comparison Group	11.32	1.07
	Intervention Group	11.50	1.25
Adaptability Post test	Comparison Group	11.68	1.08
	Intervention Group	11.53	1.28

There was a significant main effect for time in Responsibility (*p* = 0.003) and Adaptability (*p* = 0.022) skills: students’ perceptions were significantly higher in post than pretest. There was no significant main effect for group in none of the skills.

### Results from teachers’ perceptions

Results indicated a significant interaction between the effects of time and group for almost every skill: Creativity (*F*(1, 94) = 70.90, *p* < 0.001, partial Ƞ^2^ = 0.43); Cooperation (F(1, 94) = 19.95, *p* < 0.001, partial Ƞ^2^ = 0.18); Persistence (F(1, 94) = 36.32, *p* < 0.001, partial Ƞ^2^ = 0.28); Responsibility (F(1, 94) = 8.45, *p* = 0.005, partial Ƞ^2^ = 0.08); Adaptability (F(1, 94) = 8.13, *p* = 0.005, partial Ƞ^2^ = 0.08); and Curiosity (F(1, 94) = 5.74, *p* = 0.019, partial Ƞ^2^ = 0.06). Table 3 shows that for Creativity and Persistence, teachers’ perceptions in the intervention group increased in the post test, while in the comparison group decreased. We observed the opposite for Cooperation and for Responsibility, Adaptability and Curiosity teachers’ perceptions from both groups increased in the post test.

**Table 3:** Descriptive statistics on SESS results, teachers’ version.

Socio-emotional Skill	Group	*M*	SD
Creativity Pretest	Comparison Group	3.70	0.78
	Intervention Group	3.46	0.56
Creativity Post test	Comparison Group	3.44	0.67
	Intervention Group	3.95	0.74
Persistence Pretest	Comparison Group	4.13	0.93
	Intervention Group	3.81	0.69
Persistence Post test	Comparison Group	3.90	0.81
	Intervention Group	4.31	0.76
Cooperation Pretest	Comparison Group	3.95	0.79
	Intervention Group	4.13	0.52
Cooperation Post test	Comparison Group	4.22	0.59
	Intervention Group	3.85	0.65
Optimism Pretest	Comparison Group	4.13	0.64
	Intervention Group	4.12	0.45
Optimism Post test	Comparison Group	4.21	0.47
	Intervention Group	4.25	0.38
Responsibility Pretest	Comparison Group	3.92	0.67
	Intervention Group	3.57	0.62
Responsibility Post test	Comparison Group	4.12	0.66
	Intervention Group	4.02	0.80
Curiosity Pretest	Comparison Group	4.49	0.56
	Intervention Group	3.83	0.53
Curiosity Post test	Comparison Group	4.67	0.39
	Intervention Group	4.22	0.60
Adaptability Pretest	Comparison Group	12.55	1.65
	Intervention Group	11.52	1.33
Adaptability Post test	Comparison Group	13.00	1.37
	Intervention Group	12.49	1.50

There was a significant main effect for time in Curiosity (*p* < 0.001); Responsibility (*p* < 0.001); Adaptability (*p* < 0.001); Creativity (*p* = 0.009); Optimism (*p* = 0.009) and Persistence (*p* = 0.031). For all of these, teachers’ perceptions were significantly higher in post than pretest. There was a significant main effect for group in Curiosity (*p* < 0.001) and Adaptability (*p* = 0.008): teachers’ perceptions were significantly higher in the comparison group than in the intervention group.

### Dosage data

On average, each class had one seminar on introduction to radio, 7 lessons on well-being, 10.5 script writing lessons, 15.7 recording sessions and 2 sound design lessons. Students’ participation rate was in average, 93% and they have recorded 58 radio shows.

### Correlation between dosage and outcomes

Pearson analyses shows a significant positive correlation between Dosage and Responsibility, [*r* (73) = 0.26, *p* = 0.029], Curiosity [*r* (73) = 0.24, *p* = 0.038], Persistence [*r* (73) = 0.27, *p* = 0.020] and Adaptability [*r* (73) = 0.25, *p* = 0.036] perceived by teachers in the post test. There was no significant correlation between dosage and self-perceived skills by students.

### Students’ responsiveness

Eighty-two out of 92 students prefer the recording sessions (53 responses), followed by lessons on well-being and citizenship (33) and finally, script writing lessons (16). They considered that the academy was interesting (3.9 out of 5 points) and useful (3.5 out of 5 points).

On lessons learned, eight themes emerged (*N* = 81): (a) socio-emotional skills (e.g., teamwork, communication); (b) learning about themselves (e.g., strengths, skills, personal interests); (c) learning about their colleagues; (d) technical skills (e.g., script writing, recording); (e) character strengths; (f) thoughts/ perspective on things; (g) how radio operates; and (h) other factual learning (e.g., about people, professions). Table 4 shows the number of references and examples for each category. Six students answered that they have not learned anything or that they did not know.

**Table 4:** Learning reported by students: categories, number of references and examples (*N* = 81).

Categories	References	Examples
Improving socio-emotional skills (e.g., teamwork, communication)	twenty-nine	*“I strengthened my ability to work as a team, my ability to concentrate and learned to share leadership.”*
Learning about themselves (e.g., strengths, skills, personal interests)	twenty-four	*“In the first sessions I was able to learn more about myself and my colleagues and reflect on my ability and skills.”*
Learning about their colleagues	seventeen	*“I learned more about my group mates, and so nowadays we get along better.”*
Improving technical skills (e.g., script writing, recording)	fifteen	*“I learned how to develop a theme and how to make a script.”*
Learning about character strengths (e.g., what they are, their importance)	thirteen	*“I learned that we all have character strengths, some are well developed but the others need a little more work.”*
Thoughts, perspective on things	ten	*“I learned that we see ourselves in a different way from other people.”*
Learning about how radio operates	nine	*“I learned more about radio, since nowadays my generation does not use it as much.”*
Other factual learning (e.g., about people, professions)	seven	*“I learned a lot from the scripts, because we developed topics about which I did not have much knowledge and we did a very interesting interview that brought me a lot of information*.”

Another, more subjective survey asked the 9th grade participants how they were experiencing the academy. From the 66 answers, three main points stand out. The first one was the positive feelings about the academy:

*“This project brings me a lot of energy, enthusiasm and joy!”*

The learning/ improving of socio-emotional skills was another:

*“… I have also learned to improve my ability to speak in audiences with more people.”*

Finally, the feeling of freedom to choose the themes was very appreciated by students:

*“I feel a sense of freedom when I am able to say what I want, because we have to develop the subject a lot.”*

## Discussion

The data interpretation and discussion that follows should be read with caution, since the two groups are not comparable in some of the variables.

We found significant increases in the teachers’ perceptions of student’s socio-emotional skills namely, creativity, persistence, responsibility, adaptability, and curiosity, by the end of the academy. When analyzing these results, we must consider the main effect of time, which is not a surprise, given the “normal” youth development. Nevertheless, teachers’ perceptions of student’s creativity and persistence only increased for the students in the intervention group, which suggests a positive relation between the participation in the academy and the development of such skills. These findings corroborate that socio-emotional skills can be shaped through learning (Kautz et al., 2014; Gueldner et al., 2020). Some authors suggest that instruction, practice, and feedback may be the most important elements for promoting socio-emotional skills (Gueldner et al., 2020; Danner et al., 2021). The academy staff and some teachers highlighted the opportunity that students had to review and improve their scripts after feedback had been given. One of the teachers (of Portuguese Language) mentioned that this training allowed the students to improve their written expression. Based on students’ responsiveness, we hypothesize that the opportunities for rehearsing and recording the radio shows might have been an important feature to improve student’s oral communication.

One aspect that may have contributed to the effectiveness of the academy was the positive climate of the classroom. According to Durlak and DuPre (2008, p.337) the “positive work climate” is an organizational specific factor that affects program implementation. From the qualitative analyses, we know that students associated positive emotions—“energy, enthusiasm and joy”—to the academy, which we believe has contributed to maintain their motivation, especially in the most challenging moments (e.g., scripts writing and covid confinement).

Results show that students’ involvement in the academy (dosage) was positively correlated with teachers’ perception of students’ responsibility, curiosity, persistence, and adaptability. This can be interpreted in two ways: teachers’ perception was positively influenced by students’ attendance and participation in the sessions, or the more students participated, the more teachers were able to observe their progress. Research shows that dosage is related to the efficacy of socio-emotional development programs (Durlak, 2016; Rojas-Andrade and Bahamondes, 2019), so possibly students’ involvement has contributed to their socio-emotional skills’ development.

Teachers gave worse ratings to students’ ability to cooperate by the end of the academy. This may have happened because prior to the academy, teachers had fewer opportunities to observe their students engaging in cooperative work and, thus a more optimistic perception was held by teachers at the beginning of the school year. We also realized that students did not have training in group working, prior to the academy. Teamwork is not a widespread approach in Portuguese middle and high school education system. Another explanation may lie in the fatigue that students might have felt at the end of the school year and that may have affected their tolerance and ability to work in a more cooperative fashion.

In post-test results, participants either perceived themselves worse or there were no significant differences for curiosity and adaptability, compared with self-perceptions of students from the comparison group. An explanatory hypothesis may be the “John Henry” effect, as mentioned in the literature, in which the comparison group seeks to compensate for not being part of the project with an extra effort to develop these skills (Murnane and Willett, 2010).

It is worth mentioning that this study used self-reported measures, reflecting adolescent’s perceptions of their own skills, based on their knowledge of themselves at a given moment in their lives, thus influenced by biases (Vazire and Carlson, 2011) and developmental factors (Soto et al., 2021). The evaluation outcomes from 34 academies under the major program *Gulbenkian Academies of Knowledge (AGC),* where *Semear Valores On-air* was included, also revealed that teachers and professionals were the ones reporting the greatest changes in participants’ socio-emotional skills, while children and adolescents reported minor changes (Castro et al., 2022). These results led the AGC evaluation team to question if the self-awareness gained during the project would be responsible for these findings in students’ perceptions (Castro et al., 2022). It may have happened that, before participating in the academy, students were less aware of their skills’ level, which led them to formulate a less realistic perception of their own skills, compared to the end of the project. Students’ feedback highlighted an increased awareness of their skills and personal strengths. Adolescents also mentioned that they improved some socio-emotional skills, like teamwork, despite quantitative analysis not showing any improvement in cooperation skills. This hypothesis of overestimation of competencies is in line with a recent OECD (2022) study which shows that Portuguese adolescents, compared to the international average, reported a higher skill level in more skills than children, namely, in collaboration (of which cooperation is a subscale). To overcome this limitation, Soto et al. (2021) suggest more comprehensive forms to evaluate socio-emotional skills, like performance-based assessments for specific skills (e.g., *creativity*; Torrance, 1966), or situational judgment tests where hypothetical scenarios calling for certain skills are presented and the effectiveness of individuals’ selected responses are graded (e.g., *emotion regulation*; MacCann and Roberts, 2008). The use of behavioral checklists and rating scales (e.g., *social skills*, Goldstein and McGinnis, 1997) is also a complementary way to assess socio-emotional skills not prone to participants’ biases.

Dosage data, meaning the degree of students’ attendance and participation in sessions was very high. We believe that that happened for two main reasons. The first is that the academy was integrated in class/ school curriculum plus, the main teacher was an ally of the team, meaning students had to attend the sessions. However, the number of radio shows that each group created and recorded was entirely dependent on students’ motivation and that number met or exceeded expectations. There are not many studies comparing mandatory versus voluntary participation in SEL programs (e.g., an exception is Meyer et al., 2019), but we know that *whole school approaches* are more effective (Durlak and DuPre, 2008; Dobia et al., 2020). A second reason may be the appealing methods and activities proposed, which allowed students to be heard, to create, to get to know each other better and oneself, and to be positive agents in their communities. Several authors (Figueiredo, 2017; Frasquilho et al., 2018; Dobia et al., 2020) advocate for the importance of creating such opportunities to engage students in universal SEL programs.

We must consider some limitations of the present study. Firstly, the instruments used to assess socio-emotional skills measured respondents’ perceptions, thus subjected to biases and developmental factors. The academy’s evaluation would have benefited from more objective measures, such as the observation and recording of the participants’ behavior related to communication and other socio-emotional skills, during some specific assignments. The second limitation is not having done a follow-up assessment to see if changes sustained in time. Finally, although it was not our goal to study the relationship between socio-emotional skills and other dimensions, the project would have benefited from collecting students’ grades and comparing them with student’s socio-emotional skills.

We believe that the present paper contributes to the design and implementation of SEL programs, by (1) showing how the model of character strengths and virtues (Peterson and Seligman, 2004) can inform a SEL project, (2) how one can use project pedagogy focused on the creation of a radio to help adolescents develop socio-emotional skills, and (3) how one can help adolescents to express themselves through their voice, making SEL programs more appealing to youth in this stage of development. It contributes to research on the SEL field, by (1) showing how implementation data adds, *per se*, important information to the project outcomes and helps to better interpret the results on socio-emotional skills, and (2) suggesting that teachers’ perceptions are more sensitive to changes in student’s socio-emotional skills.

## Conclusion

Adolescents’ socio-emotional skills can be fostered in school contexts through SEL programs, with numerous benefits. The present paper presented an online school radio SEL project and program, *Semear Valores On-air*. We analyzed (1) the association between participation in the project and the perceived skills of adolescents; (2) the association between programs’ dosage and the perceived socio-emotional skills; and (3) the participants’ qualitative feedback about the project. Our quantitative findings suggest that adolescents can be actively engaged in SEL projects and that their participation seems to be associated with modest positive outcomes in their socio-emotional skills, which seems to contrast, to some extent, to students’ qualitative feedback that highlights skills’ learning. In addition, this project shows how one can build up a SEL program based on the virtues and character strengths model combined with a project methodology that enables adolescents to create new products and to express themselves through their voice. Given the advantages of SEL programs (Durlak et al., 2011; Sklad et al., 2012; Kern and Kaufman, 2017; Taylor et al., 2017; van de Sande et al., 2019) it is important to keep monitoring the implementation of SEL interventions and to broaden the measurement of socio-emotional skills with other more objective methods. Our findings add to the previous literature (OECD, 2021) that teachers’ perceptions seem to be more sensitive to changes in students’ socio-emotional skills. In addition, it shows that participants’ involvement (dosage) and responsiveness are very important in interpreting the evaluation outcomes in a more comprehensive manner.

## Author’s note

Research has consistently shown the importance of implementing universal approaches to foster social and emotional skills in adolescents. These skills include an individual’s attitudes, internal states, approaches to tasks, management of behavior and feelings, and beliefs about the self and the world that shape social interactions. Few projects have used adolescents’ voice and the radio as a way to foster socio-emotional skills. Semear Valores On-air academy was a three-year project that aimed to develop students’ socio-emotional skills, through an online school radio, where students worked collaboratively to create radio shows and were announcers at a recording studio. The innovative curriculum motivated the students to participate, while at the same time, allowed them to develop their socio-emotional skills and empowered them as active citizens, as they reported. This article provides new ideas to stimulate the participation of adolescents that can be useful to other SEL projects and deserves further investigation. Finally, we show that even when face-to-face interaction is not possible, we can, with some adaptations, deliver the activities in creative ways that allow students to continuously develop their socio-emotional skills.

## Data availability statement

The raw data supporting the conclusions of this article will be made available by the authors, without undue reservation.

## Ethics statement

Ethical approval was not required for the study involving humans in accordance with the local legislation and institutional requirements. The studies were conducted in accordance with the local legislation and institutional requirements. Written informed consent to participate in this study was provided by the participants’ legal guardian/next of kin. Written informed consent to record the participants’ voices was also provided by the participants and the participants’ legal guardian/next of kin.

## Author contributions

All authors listed have made a substantial, direct, and intellectual contribution to the work and approved it for publication.

## Funding

The author(s) declare financial support was received for the research, authorship, and/or publication of this article.

## Conflict of interest

This study received funding from Calouste Gulbenkian Foundation (CGF), as part of the Gulbenkian Programme for Knowledge and from Municipality of Cascais. The funder CGF had the following involvement with the study: providing training and mentorship on program evaluation to the academy team and the selection of the evaluation instrument (SESS), common to all the academies that have participated. The funder Municipality of Cascais was not involved in the study design, collection, analysis, interpretation of data, the writing of this article or the decision to submit it for publication. All authors declare no other competing interests.

## Publisher’s note

All claims expressed in this article are solely those of the authors and do not necessarily represent those of their affiliated organizations, or those of the publisher, the editors and the reviewers. Any product that may be evaluated in this article, or claim that may be made by its manufacturer, is not guaranteed or endorsed by the publisher.
